# Intraoperative Enteroscopy: A Rare Case of Blue Rubber Bleb Nevus Syndrome and a Rare Complication of Cyanoacrylate Glue

**DOI:** 10.7759/cureus.58655

**Published:** 2024-04-20

**Authors:** Binura Buwaneka Wijesinghe Lekamalage, Lucinda Duncan-Were, John Llewelyn, David McGouran, Daniel Mafi, Barnaby Smith, Jeremy Rossaak

**Affiliations:** 1 General Surgery, Tauranga Hospital, Tauranga, NZL; 2 Gastroenterology, Tauranga Hospital, Tauranga, NZL

**Keywords:** upper gastrointestinal (ugi) bleeding, cyanoacrylate glue complication, blue rubber bleb nevus syndrome, midline laparotomy, upper endoscopy

## Abstract

Blue rubber bleb nevus syndrome (BRBNS) is a rare disorder characterized by venous malformations predominantly affecting the skin and gastrointestinal tract, commonly the small bowel. Small bowel gastrointestinal bleeding is often the presenting complaint and is difficult to diagnose and treat. Push enteroscopy, capsule endoscopy, and intraoperative enteroscopy are techniques described for the localization and management of small bowel bleeding. We present the case of a 68-year-old male with BRBNS who presented with symptomatic anemia and melena. Initial endoscopic evaluations identified intraluminal vascular blebs, which were injected; however, bleeding continued, prompting intraoperative enteroscopy. During the procedure, multiple small bowel vascular malformations consistent with BRBNS were identified. Cyanoacrylate glue was used endoscopically to treat active bleeding sites. The patient developed a rare postoperative complication of small bowel ischemia and obstruction secondary to cyanoacrylate glue, necessitating surgical resection. Small bowel bleeding in BRBNS poses diagnostic and therapeutic challenges. Intraoperative enteroscopy together with cyanoacrylate glue offers a valuable approach to localization and intervention. While cyanoacrylate glue is generally considered safe, rare complications, including ischemic events, have been reported. This case highlights the utility of intraoperative enteroscopy and endoscopic cyanoacrylate glue in managing small bowel bleeding associated with BRBNS. While effective, clinicians must be vigilant regarding potential complications, including ischemic events, associated with endoscopic hemostatic agents.

## Introduction

Blue rubber bleb nevus syndrome (BRBNS) is a rare condition characterized by venous malformations that form on the skin and gastrointestinal tract, particularly of the small bowel [1]. Cutaneous lesions are typically asymptomatic while gastrointestinal lesions can present with iron deficiency anemia, gastrointestinal bleeding, intussusception, or bowel obstruction [2-5]. Several treatments have been attempted for gastrointestinal bleeding due to BRBNS, including systemic therapy, endoscopy, and surgery [6]. Push enteroscopy alone in the treatment of small bowel bleeding is difficult, carries a moderate complication risk, and may be non-diagnostic [7]. Intraoperative enteroscopy is deemed the gold standard for localization and treatment of small bowel gastrointestinal bleeding [8].

## Case presentation

We present the case of a 68-year-old man referred to the hospital with a two-week history of melena and symptomatic anemia. He had a medical history of atrial fibrillation on rivaroxaban and open prostatectomy. Blood tests revealed a hemoglobin of 62g/L from the baseline of 151g/L and urea of 12mmol/L. Gastroscopy and colonoscopy failed to identify a clear cause for bleeding. Capsule endoscopy revealed multiple small bowel vascular malformations with an appearance suggestive of BRBNS. Push enteroscopy was attempted, and although few vascular lesions were injected, it was unsuccessful at controlling the bleeding. The patient proceeded to intraoperative enteroscopy.

**Figure 1:**
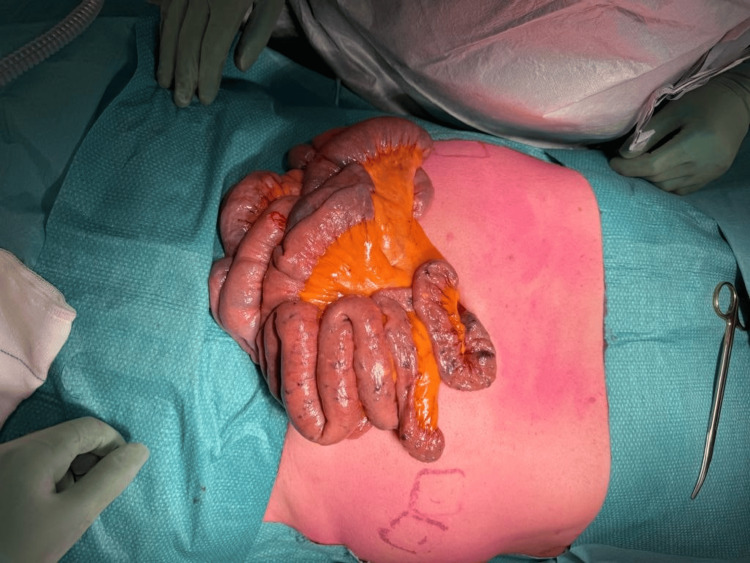
Exteriorized small bowel with vascular lesions resembling blue rubber bleb naevus syndrome

**Figure 2:**
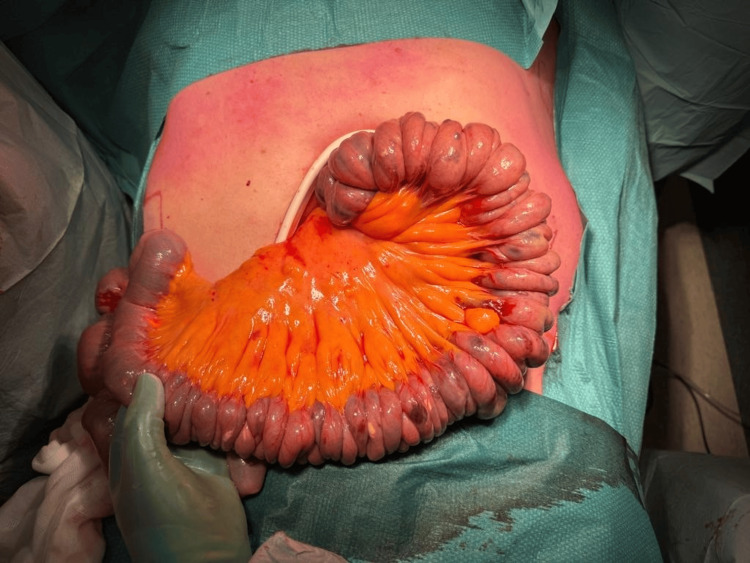
Enteroscope guided to the terminal ileum to facilitate retrograde enteroscopy

**Figure 3:**
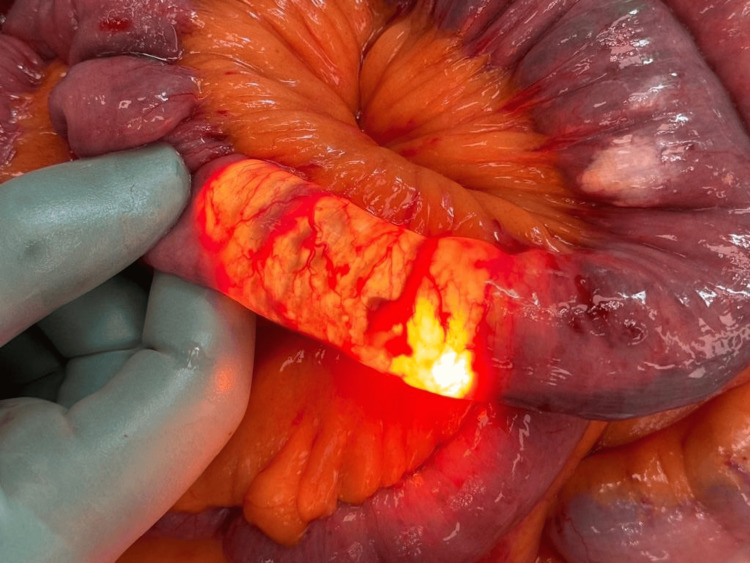
Retrograde enteroscopy with guidance from surgeon

Under general anesthesia, the surgeon first performed an upper midline laparotomy, an Alexis wound protector was utilized, and the small bowel was exteriorized (Figure 1). The endoscopist then introduced the enteroscope through the mouth and was advanced to the proximal jejunum until this could be identified by the surgeon. The surgeon guided the enteroscope through the length of the small bowel to the terminal ileum, approximately 10 centimeters from the ileocaecal valve (Figure 2). Retrograde enteroscopy was carefully performed with assistance from the surgeon (Figure 3). The entire length of the small bowel was visualized with this technique. 

**Figure 4:**
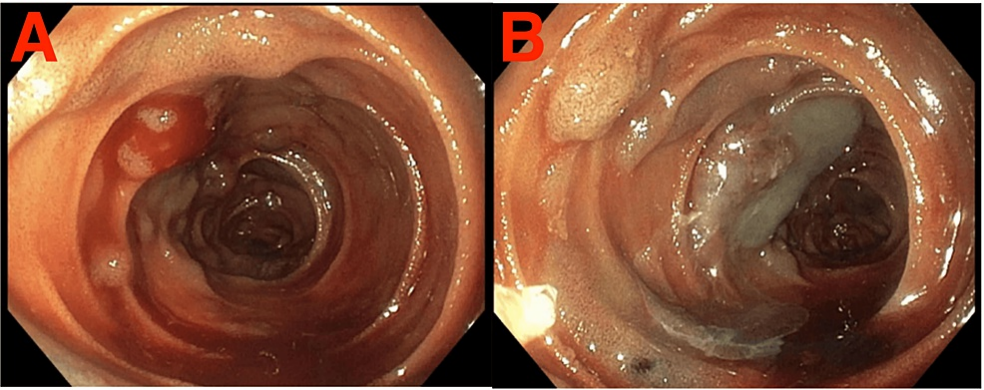
Retrograde enteroscopy performed by the endoscopist A: Endoscopic view of Intraluminal vascular lesion with active bleeding. B: Endoscopic view post treatment with cyanoacrylate glue to arrest bleeding.

Multiple "blue blebs" were visualized through the length of the small bowel (Figure 1), which correlated to the vascular lesions seen intraluminally via the endoscope. A site of active bleeding from a number of these lesions were identified endoscopically in the mid small bowel (Figure 4A). These sites were injected with cyanoacrylate endoscopic glue as a method of endoscopic hemostasis (Figure 4B). Final inspection revealed no further active bleeding from sites of injection and a total of seven milliliters of cyanoacrylate glue was used during the procedure. Once the scope was at the duodenum, it was then withdrawn, and the abdominal wound was closed. 

**Figure 5:**
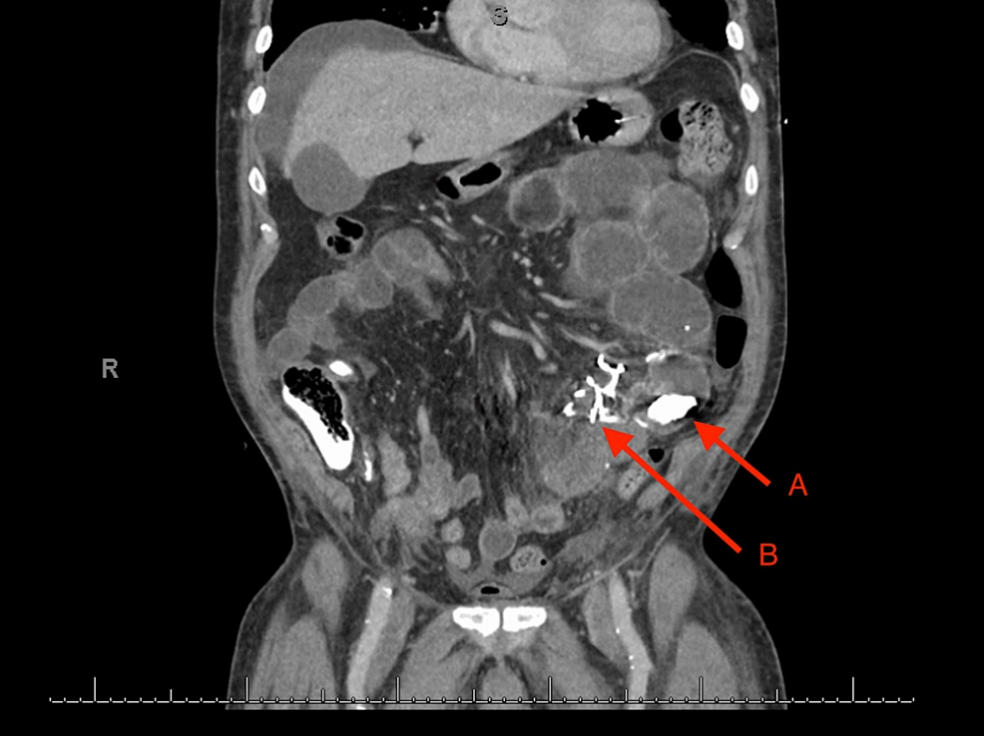
Postoperative day three arterial contrast computed tomography showing features of small bowel obstruction A: Intraluminal glue causing small bowel obstruction. B: Glue embolization into mesenteric veins.

**Figure 6:**
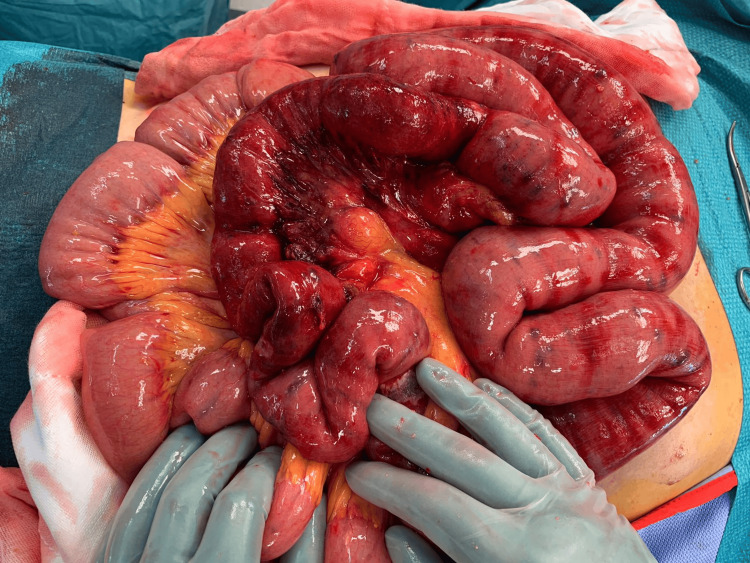
Small bowel exteriorized during exploratory laparotomy Ischemic segment of the mid-small bowel with glue tracking into the mesentery

**Figure 7:**
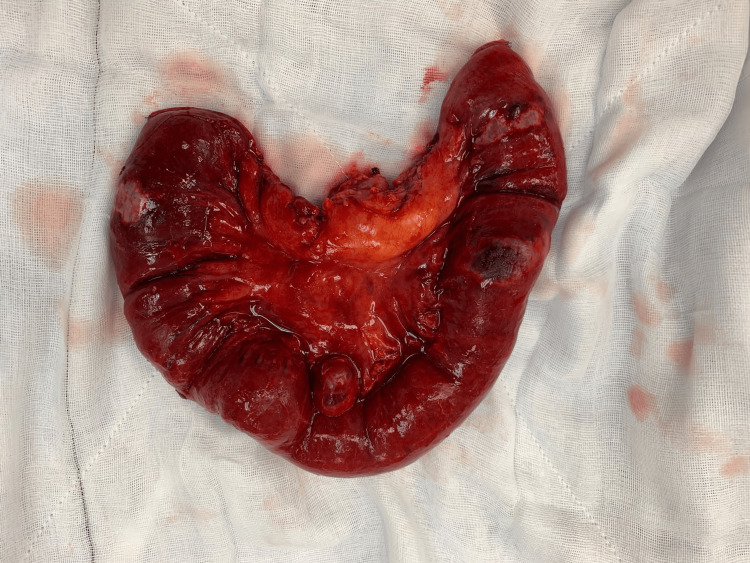
Resected segment of ischemic small bowel

The patient was returned to the ward and commenced on oral liquid diet. Three days post procedure, he became tachycardic and hypotensive. A computed tomography with arterial contrast revealed mid-small bowel obstruction from intraluminal cyanoacrylate glue and embolization of glue into mesenteric veins (Figure 5). The patient returned to the theatre for exploratory laparotomy and found 40 cm of ischemic small bowel with glue tracking through the mesentery (Figure 6). A small bowel resection and stapled side-to-side small bowel anastomosis was performed (Figure 7).

After laparotomy, the patient was extubated and returned to the intensive care unit with a nasogastric tube. His postoperative course was complicated by an ileus requiring a short period of gut rest and total parental nutrition. Diet was subsequently progressed, and the patient was discharged nine days after laparotomy. To date, he has had no further episodes of bleeding. Postoperative histology confirmed small bowel ischemia caused by venous congestion from glue embolization into the mesentery. The histology also confirmed that the small bowel vascular lesions were consistent with BRBNS.

## Discussion

Gastrointestinal bleeding from the small bowel is overall rare. Arteriovenous malformations are the most common cause of bleeding from the small bowel, such as angiodysplasia, telangiectasia, small bowel varices, and Dieulafoy lesions [7]. The treatment options are typically limited to radiological intervention, surgical resection, or push enteroscopy [6]. In our case, we outline a treatment option for small bowel bleeding due to BRBNS that permits endoscopic therapy with guidance through a mini-laparotomy incision. There have been a few documented cases where intraoperative enteroscopy has been attempted for small bowel gastrointestinal bleeding [9-11]. Overall, intraoperative enteroscopy is claimed to be 90% effective at diagnosing small bowel gastrointestinal bleeding [12]. Furthermore, it provides an option for treatment either by endoscopic hemostatic methods or surgical resection. Marking clips may also be placed intraoperatively if future intervention is required radiologically or surgically.

There are several endoscopic hemostatic methods that can be utilized in gastrointestinal bleeding, including clips, ligation, adrenaline, thermo-coagulation, and hemostatic agents [13]. When performing therapeutic endoscopy, it is important to be aware of several methods of endoscopic hemostasis. Currently, no high-powered studies exist to suggest that one method is superior, and typically a combination of methods may be administered to maximize success [14]. Cyanoacrylate glue is an established hemostatic agent for endoscopic administration. Typically, it is mixed with lipiodol to slow the rate of solidification in order to facilitate endoscopic use. Lubrication of the working channel and stopping suction are required in order to protect the endoscope [14]. Cyanoacrylate glue is effective for the treatment of gastric and esophageal varices; however, there is limited experience in other applications of non-variceal bleeding [15,16].

Cyanoacrylate has been deemed a safe method of hemostasis in endoscopy [17]. A retrospective observational study of 135 cases showed 98.5% efficacy in hemostasis and only minor complications in the endoscopic administration of cyanoacrylate glue [18]. Complications are overall uncommon; there have been two reported cases of arterial embolization with infarction in the setting of peptic ulcer disease, one being fatal [16,19]. A case of systemic embolization leading to splenic and cerebral infarction has also been reported [20]. We report a rare case of bowel ischemia and bowel obstruction due to the endoscopic administration of cyanoacrylate glue. There are no documented cases of venous ischemia and bowel obstruction as a complication of cyanoacrylate glue.

## Conclusions

Gastrointestinal bleeding from the small bowel is uncommon; it is challenging to diagnose and treat. Intraoperative enteroscopy has been described as a successful intervention for the diagnosis of small bowel bleeding and facilitates endoscopic or surgical management of bleeding. While several endoscopic methods of hemostasis exist, we describe the endoscopic administration of cyanoacrylate glue. This is typically deemed a safe and effective technique for endoscopic hemostasis, but it is important to be aware that glue can embolise, leading to ischemic effects. This case demonstrates a rare cause of small bowel bleeding due to BRBNS and a rare complication of its treatment following endoscopic cyanoacrylate administration.
